# Evaluation of Concurrent Validity between a Smartphone Self-Test Prototype and Clinical Instruments for Balance and Leg Strength

**DOI:** 10.3390/s21051765

**Published:** 2021-03-04

**Authors:** Linda Mansson, Pernilla Bäckman, Fredrik Öhberg, Jonas Sandlund, Jonas Selling, Marlene Sandlund

**Affiliations:** 1 Section of Physiotherapy, Department of Community Medicine and Rehabilitation, Umeå University, 901 87 Umeå, Sweden; linda.mansson@umu.se (L.M.); pernilla.beckman@hotmail.com (P.B.); jonas.sandlund@umu.se (J.S.); jonas.selling@umu.se (J.S.); 2 Department of Radiation Science, Umeå University, 901 87 Umeå, Sweden; fredrik.ohberg@umu.se

**Keywords:** aged, smartphone, mHealth, postural balance, muscle strength, mobile applications, validity, patient outcome assessment

## Abstract

The evolving use of sensors to objectively assess movements is a potentially valuable addition to clinical assessments. We have developed a new self-test application prototype, MyBalance, in the context of fall prevention aimed for use by older adults in order to independently assess balance and functional leg strength. The objective of this study was to investigate the new self-test application for concurrent validity between clinical instruments and variables collected with a smartphone. The prototype has two test procedures: static standing balance test in two positions, and leg strength test performed as a sit-to-stand test. Thirty-one older adults were assessed for balance and functional leg strength, in an outpatient physiotherapy setting, using seven different clinical assessments and three sensor-tests. The results show that clinical instruments and sensor measurements correlate to a higher degree for the smartphone leg strength test. For balance tests, only a few moderate correlations were seen in the Feet Together position and no significant correlations for the Semi Tandem Stance. This study served as a first step to develop a smartphone self-test application for older adults to assess functional balance at home. Further research is needed to test validity, reliability, and user-experience of this new self-test application.

## 1. Introduction

Accidental falls related to impaired balance among older adults is an increasing challenge causing suffering for the individual and substantial costs to society [1]. There is strong evidence that balance and leg strength exercises prevent falls [2]. To evaluate and follow up exercise programs and identify individuals in the risk zone, it is important to be able to measure balance and leg strength in an easy but reliable way.

The human balance system is complex and includes several motor and sensory components of postural control, e.g., motor learning, motor control, sense of stability limits, and latency to postural response [3], all affecting the ability to maintain balance. Balance contains elements of stability and orientation [4], where stability is the relation between the base of support and the centre of gravity, and orientation is the postural alignment of the body and its orientation to its surroundings.

Several clinical instruments have been developed to address the many different aspects of balance both static and dynamic balance, but no golden standard exists for clinical balance tests. Well-known examples of such instruments are, for example, the Berg Balance Scale [5] and the Mini-BESTest [6]. Other instruments have more elements of mobility in the balance assessment, like the well-established timed walking test Timed Up and Go (TUG) [7], or emphasize leg strength and endurance components, such as the 30s Chair Stand Test [8] and the Five times sit-to-stand [9,10]. Leg strength is also central to balance control and plays an important role in impeding perturbations and falls [11,12,13,14]. These standardized clinical instruments are subject to observations by the therapist, and will often also require a substantial change in balance performance before indicating a difference. Many such limitations could be reduced with the use of new technology to objectively measure different aspects of balance.

Traditionally, force plate equipment has been used for posturography to quantify postural control in terms of changes in the centre of pressure as the body sways, either during static or dynamic conditions [15,16,17,18]. However, posturography assessments require access to advanced equipment and may include elaborate protocols. An ideal balance test for widespread use in clinics, and preferably in home-settings, should be a test that is easy to administer and require minimal equipment.

Compared to clinical tests and more advanced posturography equipment wearable sensors, such as accelerometers, are accessible and often uncomplicated to use, while still having the potential to give more detailed information about seniors’ balance performance [19]. As early as 1998, accelerometer-based data were used to described to measure postural sway in static and dynamic balance tasks with uniaxial accelerometers [20], and in standing and walking with triaxial accelerometers [21,22]. Today research with inertial measurement unit (IMU) sensors to measure balance is cumulating [23,24,25,26,27,28,29,30]. IMU sensor measurements of balance have been validated against both force plate and kinematics and shown strong correlations [31,32]. The advantages of wearable sensors in posturography were described in a recent review [33] and reported as accurate, reliable, and useful, even though, as many studies are still early development studies, only 36% of the included articles described validations against a gold standard measurement. This wide range of research indicates that sensor measurements are previously tested and proven able to assess balance.

In addition, successful assessments of sit-to-stand movements with sensors have been reported [23,34,35,36,37,38,39,40] and triaxial accelerometer sensor measurements assessing standing balance have also shown correlations with clinical physiotherapy assessments for the TUG and the Berg Balance Scale [41]. The TUG test has been further developed into the instrumented version iTUG, using sensors to quantify the sit-to-stand, walking, and turning sub-components of the short mobility test [42], and smartphone technology has also been used for iTUG [43]. The iTUG is the most frequently used sensor measurement test where validation against clinical instruments have been studied. Various iTUG studies have included older adults [44,45,46,47], and research has shown that the mean angular velocity during turning and duration of the turning phase of the iTUG can predict the Mini-BESTest score [45]. Moreover, both the smartphone iTUG and the instrumented sit-to-stand test showed potential to discriminate functional ability [19], and five repeated iTUG tests have been shown to predict the Community Balance and Mobility Scale (CBMS) score [47]. These previous studies have proved sensor measurements to be valid for assessing balance function with the use of sensors in smartphones. However, several studies focused on other outcomes like duration or counting repetitions and have not only assessed body sway.

The fast development of smartphone technology has made access to high accuracy sensor measurements widely available, at relatively low costs, using familiar technology [48]. With sensors like accelerometers, gyroscopes, magnetometers, cameras etc. in smartphones today, easy non-invasive methods for monitoring of movements are readily available to benefit health promotion and healthcare [49]. Studies have shown that IMU sensors in commercially available devices like smartphones or tablets generate measures of static balance comparable to force plates [50,51,52] or external accelerometers [53]. As access to smartphones is rapidly increasing, including in the older population, it is providing opportunities to develop and distribute self-tests for balance assessments which can be used in a home-setting. This type of self-test could facilitate early detection of reduced balance as well as empower individuals to maintain fall prevention exercise. A recent usability study of a self-test application for older adults showed promising results for use in a home setting, but further improvements and validation are needed [54].

We have in co-creation with older adults developed a prototype for a self-test application, MyBalance, to be utilized independently by older adults without a health care consultation [55]. The MyBalance test measures body sway in static quiet standing and leg strength when rising from a chair, and the current study was part of the first evaluation process of this application. Information regarding the degree of validity between clinical instruments and a sensor-test would make it possible to better understand what aspects of functional balance a sensor test might reflect. In particular, the static standing balance sensor-test measures different aspects of balance than the clinical instruments do. The aim of this study was to investigate the concurrent validity between clinical instruments, and variables collected and calculated with the MyBalance prototype, for both static balance and leg strength.

## 2. Method

This observational explorative study compared a prototype self-test application, named MyBalance, with clinical instruments commonly used in the physiotherapy setting. The choice to use a smartphone application was made as ubiquitous use of smartphones is also increasing among older adults [56,57]. The application could also be integrated with self-managed fall prevention exercise programs to facilitate independent follow-up measurements. MyBalance was developed in co-creation with older adult users to improve user experience [55].

Association between the prototype’s variables from a Three Maximal Chair Stand Test and a static standing balance test in two positions (Feet Together and Semi Tandem), and corresponding clinical instruments was analysed. The first evaluation was made comparing the self-test application to commonly used clinical instruments, as this is more relevant for physiotherapists in their assessments, rather than validate it against gold standard lab equipment. The MyBalance prototype was developed by engineers in close collaboration with physiotherapists.

### 2.1. Participants

Thirty-one participants (24 women and 7 men) with a mean age of 79 years participated in the study and were all recruited in a primary health care setting through convenience sampling. Older adults who met healthcare rehab staff were asked to participate and were given both verbal and written information. Interested individuals were contacted by the test leader for further information about the study, and an appointment for the test procedure was arranged. Inclusion criteria were: ≥70 years old, able to rise from a chair independently, community-dwelling, and able to understand and read Swedish. Any self-reported progressive disease that was likely to influence cognitive function, or any impaired cognitive function judged by the test leader during the individual information session, was cause for exclusion. The first 21 participants were tested in one clinic and the remaining 10 in a different clinic. The study was approved by the Regional Ethical Review Board, Umeå, Sweden (dnr 2017/317-31) and written informed consent was collected from participants.

### 2.2. Data Collection

**Figure 1:**
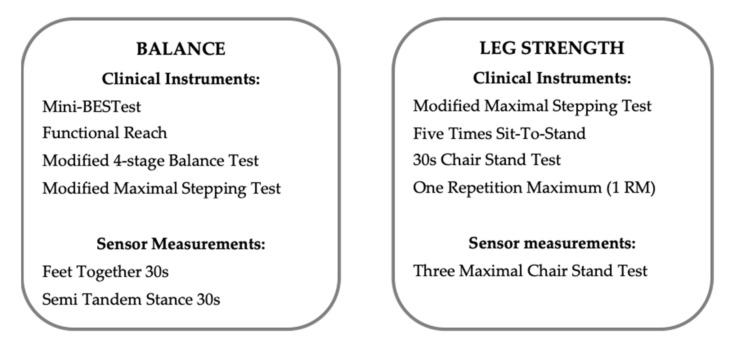
Clinical tests for balance and functional leg strength as well as sensor measurements with the MyBalance prototype used in this study.

The data collection was made by an experienced physiotherapist (PB) using a set of balance and functional leg strength tests (Figure 1), of which two were performed with the MyBalance prototype. A questionnaire was completed to provide background data of participants’ self-reported medical conditions, previous accidental falls, as well as level of physical activity scored by the Saltin-Grimby Physical Activity Level Scale (SGPALS), which also assimilates household activities [58]. The SGPALS was dichotomized into inactive (level 1–2) and active (level 3–6) to describe the level of physical activity. In order to calculate sensor measurement variables correctly, participants height and weight were measured.

#### 2.2.1. Clinical Instruments for Balance

The following four clinical balance instruments were used in this study. The variety of instruments was based on their ability to assess different aspects of balance:

(1) Mini-BESTest (MiniB) [6] is considered to be a reliable [59] and valid test [60] that evaluates balance in different dimensions (anticipatory, reactive postural control, sensory orientation, and dynamic gait) with 14 different tasks. Score range is 0–28 points and a higher score indicates better balance [6].
(2) Functional Reach (FR) is a reliable [61] and valid test [62] that measures postural control. Participants are instructed to lean forward as far as possible, with an extended arm at 90 degrees shoulder flexion parallel to a wall, and at the same time retain their foot position. The result is measured in centimetres. After two practice runs, three trials were performed and the mean value was used in the analysis.
(3) The Modified 4-stage Balance Test (m4-stageBT) evaluates balance by testing four progressive balance positions: Feet Together, Semi Tandem Stance, Tandem Stance, and one leg stand. A modification, from the original 4-stage Balance Test [63] was that each position would be held for 30 s (in the original test 10 s), and that the total time in seconds was used in the analysis (max 120 s). According to instructions for the 4-stage balance test, it was optional to hold on to a support to get into the start position, and a complete test was required to try the subsequent position [63].
(4) The Modified Maximal Stepping test (MaxStep) [64] evaluates how far a person can safely take a step and is considered to be able to predict future falls. This test was modified from a more advanced stepping test [65]. Participants were instructed to take one step forward, along a measured line, and then return to the starting position. The result was measured in centimetres and the use of either foot was permitted. After two practice runs, three trials were performed and the best result of the three was used in the analysis.

#### 2.2.2. Clinical Instruments for Functional Leg Strength

The following four clinical leg strength instruments were used in this study:

(1) The Modified Maximal Stepping test (MaxStep) was used both as a balance instrument and a leg strength instrument.
(2) Five Times Sit-To-Stand (5TSTS) [10] is both a reliable [66] and valid test [9] for measuring function in the lower extremity. It is performed by doing five chair stands from a normal height chair, with arms crossed over the chest. The result is measured in seconds and shorter duration indicates better test performance. The test was performed twice and the mean value was used in the analysis.
(3) 30s Chair Stand Test (30s CST) is a reliable and valid test [8] to measure muscle function in the lower extremity. Instructions were to stand up from a normal height chair as many times as possible during 30 s, with arms crossed over the chest.
(4) One Repetition Maximum in sitting leg press (1 RM) is a reliable and valid test to measure older people’s leg strength [67,68]. The test was performed in a way similar to the one carried out in the study by Hasselgren et al. [67]. Five repetitions with low resistance were performed as a warm up and the participant was instructed to fully extend their knees. If the participant could rise from a chair (45 cm) with arms crossed over the chest, the test was then started at a weight as close to 10 kg below bodyweight as possible. If not possible, then the test was started at 10 kg below half the bodyweight. To start, the participant was seated with a 90-degree knee flexion. An increase of nine kilograms with a 45 s rest between each repetition was done until the participant did not manage to complete the full leg press movement. The maximum weight was recorded as the person’s 1 RM. In this study, Life Fitness equipment was used for the seated leg press.

#### 2.2.3. Sensor Measurements Performed with the MyBalance Prototype

Sensor data collection was done with the MyBalance prototype on an Android smartphone (Sony Xperia X Compact F5321) connected via Bluetooth to a PC to register acceleration from body movement. The smartphone was placed in an upright position and attached to the lower back (L4–5) around the waist, using a sports armband with an elastic Velcro band as an extension. The custom Android application was developed to sample the built-in gyroscope and accelerometer data at a frame rate of 100 Hz, and then transmit the data in real time (a package of data every half second) to a custom MATLAB application on the computer for storage and later analysis.

Three assessments with the MyBalance prototype were completed in this study:

(1) Feet Together, (static standing balance test): on a given signal from the test leader, the participant was instructed to maintain balance, with their feet positioned close together for 30 s. If a support to get into the start position was used, the person let go and thereafter the timer started. The Feet Together position was repeated three times to gain more reliable data collection. It was only repeated for the easiest test position so as not to challenge the participants extensively. It coincided with the first position in the clinical assessment Modified 4-stage Balance Test.
(2) Semi Tandem Stance (heel beside the big toe on the other foot), (static standing balance test): on a given signal from the test leader, the participant was instructed to maintain balance, with their feet standing in the semi tandem position for 30 s. The option for support to get into the start position was available with the same conditions as for Feet Together. This position coincided with the second position in the clinical assessment Modified 4-stage Balance Test.
(3) Three Maximal Chair Stand Test, (leg strength test): this is a modification of the traditional Five Times Sit-To-Stand test previously used in sensor measurements to calculate muscle power [39]. The Three Maximal Chair Stand Test was performed from a sitting position on a chair of normal height. On the command from the test leader (stand up and sit down), the participant was instructed to rise from the chair as fast as possible, with arms crossed over the chest, and then stand still until the test leader asked the person to sit down. Between each movement, a pause for at least three seconds was held before the next rising or sitting movement. The test included two practice runs, followed by the Three Maximal trials, mean value was used in the analysis.

### 2.3. Test Procedure

Each participant attended one test session lasting about one hour. Clinical tests were performed according to test manuals for each test, and the physiotherapist was familiar with the instruments. Tests were performed in the same sequence for all participants: Background questionnaire, Mini-BESTest, Functional Reach, Modified 4-stage Balance Test (including sensor-tests: Feet Together and Semi Tandem Stance), Three Maximal Chair Stand (sensor-test), Five Times Sit-To-Stand, 30 s Chair Stand Test, Modified Maximal Stepping test, last part of the questionnaire, and 1 RM. To provide a short rest before the last clinical test (1 RM), a pause was scheduled and participants filled out the last third part of the background questionnaire. The sequence of the tests was decided to facilitate the two sensor-tests running one after the other (one fitting of the smartphone), as well as to end with the most demanding strength test. As the testing procedure was done in two different clinics, two different types of Sitting Leg Press Life Fitness equipment were used. Due to this change in test condition, only the first 21 participants were included in the analysis of 1 RM.

### 2.4. Sensor Measurement Data Processing

The following two steps were undertaken for data processing: (1) Data were lowpass filtered at half sampling frequency with a 4th order zero-phase Butterworth filter (MATLAB filtfilt). (2) To compensate for pelvis tilt and rotation, the orientation of the smartphone was derived by a quaternion-based orientation filter [69]. The filter uses the accelerometer and gyroscope data to estimate the orientation of the smartphone at every frame, which is then used to transform the raw accelerometer data to purely vertical and horizontal components. Variables for the balance test were derived from the horizontally transformed accelerometer data. Variables for the leg strength test were derived from the vertically transformed accelerometer data. See Table 1 for the four leg strength variables and eight balance variables.

The procedure to set events and intervals manually was performed in the following way. With regard to the standing balance tasks, Feet Together and Semi Tandem Stance, intervals were analysed for one second after timer start (to exclude initial arm motions) until timer stop, resulting in 29 s of analysed data. For the leg strength test, Three Maximal Chair Stand Test, the interval of each rising motion was manually identified in MATLAB by selecting a still period (low accelerometer activity) just before and after each rise from the chair. Within this given interval, the start of the motion was automatically identified based on the average acceleration one second prior to the interval. The start event was placed at the last acceleration minima, prior to the time at which acceleration rose 0.5% above the previous mentioned average. The end of the rising motion was defined as the time point after maximum deceleration in the interval where acceleration crossed 0.5% below previous mentioned average.

### 2.5. Data Analysis 

For variables with more than one attempt (Five Times Sit-To-Stand, Feet Together and Three Maximal Chair Stand Test), a mean value was calculated. For the Functional Reach and Modified Maximal Stepping test, a normalization variable was calculated before statistical analyses. This was done by dividing the measured value with the height of the individual. The Modified Maximal Stepping test correlated with both balance and leg strength instruments and was therefore assigned to all sensor measurement correlations. For 1 RM, the relative strength was used in the analysis, dividing the maximum weight from the leg press with the body weight. The distribution of the data was examined and found to be normally distributed for all groups of variables, with the exception of the time domain variables in the balance sensor-test. However, as several clinical instruments provided ordinal data, non-parametric correlations were used in all analyses. Descriptive data for the tests were presented to show distribution. For balance measurements, correlation analyses were carried out to illustrate the relationship between both the scores from the clinical balance instruments with the MyBalance prototype and also the variables from Feet Together and Semi Tandem Stance sensor measurements with the MyBalance prototype. Correlations were also calculated between results from the functional leg strength clinical instruments with the variables from the Three Maximal Chair Stand Test sensor measurements. A selection of sensor variables was used in order to have variables representing different dimensions of balance. Correlations were calculated with the Spearman’s rank correlations coefficient, as some of the clinical instruments had an ordinary scale. For correlation, the following levels were applied: very high correlation (0.90 to 1.00), high correlation (0.70 to 0.90), moderate correlation (0.50 to 0.70), low correlation (0.30 to 0.50), or poor correlation (less than 0.30) [71]. A negative correlation value is generated if one variable increases while the other decreases, and vice-versa. In this study, a lower value indicates a better function for 5TSTS and balance time domain variables. The level of significance was set to *p* < 0.05. The analyses were done using jamovi version 1.1.9.0, the jamovi project (2020) [Computer Software].

## 3. Results

The following results section contains the descriptive data for the group of individuals that participated in this explorative study. Moreover, results of the correlation analyses are presented. Firstly, between the scores from the clinical instruments with each other and secondly, between the scores from the clinical instruments and the sensor measurements, in relation to both the balance tests and the leg strength test.

### 3.1. Descriptive Data

Descriptive data concerning the 31 participants and data from the clinical instruments are summarized in Table 2. About one-third of participants reported a fall during the last 12 months and the group was considered fairly active. The same numbers of participants are noted as active for summer and winter but do not represent the same individuals. For the Modified 4-stage Balance Test, a ceiling effect was noted, where the median score was in the upper third of the scale.

### 3.2. Correlation between Clinical Instruments

The four balance instruments used showed moderate to high correlations between each other. The four leg strength instruments showed a weaker correlation in general, with the only high correlation between the Five Times Sit-To-Stand and 30s Chair Stand Test. The Modified Maximal stepping test was the only leg strength instrument with significant correlation to all the other leg strength instruments. The Modified Maximal stepping test also correlated with all balance and leg strength instruments. All correlation data are presented in Table 3.

### 3.3. Correlation between Sensor Measurements

The complete correlation analyses can be found in Supplementary Figure S1A,B for all measured variables. In this article, we have chosen to describe eight selected balance variables and four leg strength variables, as previously mentioned in the Method section. In summary, the time domain variables for balance, in both foot positions, showed very high correlations between each other. For frequency variables as well as leg strength variables, the correlations were more mixed, including moderate to very high correlations.

### 3.4. Balance Sensor-Tests and Correlations with Clinical Instruments

Data from the smartphone sensor measurements for Feet Together and Semi Tandem are presented in Table 4.

The main results of the correlation analysis between the clinical balance instruments and the sensor-test variables for Feet Together are presented in Table 5. The frequency variables in the medio—lateral direction showed moderate correlations with most clinical instruments, whereas correlations between the anterior–posterior direction and most clinical instruments were poor. For the time domain variables, only Mini-BESTest and medio–lateral P2P showed a significant, although, low correlation. No significant correlations were observed between the Semi Tandem Stance and the clinical instruments (Table 6).

### 3.5. Leg Strength Sensor-Test and Correlations with Clinical Instruments

Data from the sensor measurements taken with the smartphone for the Three Maximal Chair Stand Test is presented in Table 7.

All smartphone variables for the Three Maximal Chair Stand Test correlated significantly with both the Five Times Sit-To-Stand test and the 30s Chair Stand Test, ranging from low to moderate. All variables except PowerMax showed low to moderate correlation with the Maximal stepping test. Moreover, only PowerMax and VelMax showed significant moderate correlations with 1 RM in sitting leg press. All data from the correlation analysis can be found in Table 8.

## 4. Discussion

The results from this study show that clinical instruments and the sensor measurements had significant low to moderate correlations for the leg strength tests, but poorer correlations for the balance tests. In the standing balance sensor test, for Feet Together, correlations were in general poor, but for frequency variables in the medio—lateral direction, low to moderate correlations were seen with all clinical instruments. For the Semi Tandem Stance, however, no significant correlations with the clinical instruments were seen. The leg strength sensor variables showed low to moderate correlations for most comparisons with the clinical instruments, indicating acceptable concurrent validity for leg strength with the MyBalance prototype.

The low level of correlations between clinical instruments and the balance sensor variables implies that different aspects of balance are assessed in the different types of tests. A physiotherapy assessment is undoubtedly distinct from a sensor measurement of body sway in static standing. The clinical balance assessments evaluate the ability to maintain equilibrium during different tasks, and assess the individual’s postural control in interaction between the environment and the postural task, in both static and dynamic conditions [4]. Thus, clinical balance tests assess a variety of different aspects of balance, in contrast to static balance performance. For example, the Functional Reach test, assesses postural control limits of stability [61] and the Mini-BESTest and Modified Maximal Stepping test both include aspects of dynamic balance. IMU sensors on the other hand measure accelerations (rate of change of velocity) [21], and can therefore register the ability to stay steady; a static balance performance which could be challenging to detect with the human eye. In addition, sensor measures have in general better metric properties compared to clinical tests, as they can measure more accurately, e.g., duration and angular velocity [45]. Considering that balance is complex, including many different aspects and dimensions, low correlations between static sensor-tests and clinical instruments that measure many aspects of functional balance are not surprising and are in line with observations in previous research [72]. In fact, the clinical instruments in our study in general also only showed moderate correlations with each other, which has also been reported previously [73].

With accelerometery, like force plate posturography, there is no agreement about which variables should be used in the assessment of postural control—an infinite number of variables can be extracted and the interpretation in relation to specific control mechanisms, postures, and clinical characteristics is a matter of debate [4,72]. Nevertheless, in our study, the frequency domain variables showed stronger correlations to all clinical tests than, the more traditionally used, time domain variables indicating clinical validity, specifically in the medio–lateral direction in the Feet Together position. The positively directed correlations imply better balance scores in clinical tests with higher median and centroid frequencies of the sway spectra. High frequencies are indicative of faster and smaller postural adjustments and low frequencies of slower and larger adjustments that may serve different purposes in the control of balance [74,75]. It was, however, beyond the scope of the present study to speculate on different mechanisms and control strategies employed by the participants. The results are, however, in accordance with previous studies. Which have suggested that validity of sway frequency features and, possibly, better overall balance ability, in comparison with other estimates are able to discriminate between older and younger subjects, and different postural demands [23,26]. Interestingly, a recent study with older adults showed that lower frequency variables from force plate measurements in static standing related to a higher fall related concern and decline in sensory and motor function [76]. The interest in the frequency analysis of postural control is increasing, although the interpretation of specific frequency bands may not be straightforward [75]. Future studies may, however, as suggested by Moe-Nilsen and Helbostad [23], want to investigate further to what degree different sway frequency variables, and the theories and interpretations suggested in force-plate research, are relevant to the acceleration signal at the level of the lower spine that estimates centre of mass [74].

Regarding the standing balance sensor-tests, interestingly, the Feet Together position yielded more correlations to the clinical tests compared to the more challenging Semi Tandem Stance position. Similar results were seen for balance assessments, using wireless skin-mounted sensors, in both persons with multiple sclerosis and healthy participants, indicating that more advanced balance positions were not necessary to distinguish reduced balance [77]. The poor correlation between clinical tests and sensor measurements in the Semi Tandem position in our study could possibly be explained by the rather unnatural position. Arguably, tandem and semi tandem positions are uncommon in everyday situations, and may invoke more exploration and randomly organized sway as subjects search for an appropriate balance strategy. As positions and support surface influence the postural sway, a standardized protocol for foot positions may be required to enable comparison between studies [33]. An alternative suggestion was that a self-selected foot position might be the most suitable for assessment outside a clinical setting to get a measurement closer to real life situations [33]. Further investigations are clearly needed on the influence of foot positions on balance sensor variables when developing self-assessment tools for smartphone devices.

Regarding results on the leg strength sensor-tests, our results corroborate the findings of previous studies. Regterschot et al. [39] found that sensor measurements could accurately assess leg strength and power, and showed higher sensitivity for improvements compared to standard clinical assessments. Likewise, measurements with a smartphone for the 30 s Chair Stand Test have shown potential to discover functional decline in healthy older adults [19]. In younger adults wearing smartphones in the pocket, good correlations were reported for the TUG, 30 s CST and 5STST for measures of duration and number of repetitions [78].

Some methodological considerations of the present study should be addressed. The smartphone sensor registrations were not done concurrently with the clinical instruments, except for the Modified 4-stage Balance Test and balance sensor-test. This may limit the correlation strength between the tests. Even though the balance sensor-test was performed at the same time as the Modified 4-stage Balance Test, Feet Together and the Semi Tandem Stance, the correlation was limited—likely, at least in part caused by the observed ceiling effect of this clinical test. Further, the same type of leg press equipment was not available at the second clinic where data collection took place, and results for the 1 RM test for 10 participants, unfortunately, had to be excluded from the analysis. A positive point was that the prolonged standing balance time to 30 s (from the original 10) was manageable for the participants. The prolongation was primarily done to get a more adequate sensor measurement for each position by extending the time of data collection [79]. Further, no adverse events were noted during data collection, and it was encouraging that the test performance for the sensor measurements in general was considered safe to perform by participants. Another positive point was that no technical problem was reported.

Regardless of the limited correlations with clinical instruments, the prototype may still be accurate as a sensor measurement and a valuable complement to clinical assessments. Two previous review studies have described the ability to use sensor measurements to assess balance [33,80]. In addition, the algorithms have previously been evaluated and found valid [25,34,40]. The MyBalance self-test application could offer an easy, accessible and useful tool for assessing balance, to be used by older adults at home without health care consultations and provide feedback and follow-up over time. However, more validity testing is required using gold standard measurements to confirm the new self-test applications validity, as well as affirm reliability. The remote monitoring, and potential for early detection of change in function are also appealing benefits offered by novel smartphone applications [78]. Balance assessments from wearable sensors have been shown to correspond with clinical fall risk assessments [81]. It could therefore offer a quick and objective balance assessment for physiotherapists and other healthcare workers, to be used as a complement to current assessments. However, the intention with the MyBalance application is primarily to increase motivation to perform fall prevention exercise. We do not intend to provide a complete fall risk assessment application but facilitate self-monitoring by tracking deterioration or improvement of balance function while engaging in fall prevention interventions.

## 5. Conclusions

Sensor measurements from the smartphone application prototype showed a higher degree of correlation for the leg strength test than for the balance tests when compared to clinical instrument assessments. In the Feet Together position a moderate correlation was seen only for frequency variables in the medio–lateral direction and clinical balance instruments. In the Semi Tandem Stance, no correlations were seen with clinical instruments. Significant low to moderate correlations were seen for most leg strength sensor variables compared to the clinical instruments. Additional studies are required to validate the MyBalance self-test application with some of the golden standards such as force plate, movement sensor system, etc. Moreover, a user-test of the self-test application MyBalance is necessary to evaluate the usability for the older population. Reliability test-retest of the application is also needed before deployment. Our results show clinical validity of leg strength assessments and frequency variables of postural sway while standing. As noted, it is unlikely that assessing only leg strength and body sway in static standing would provide all the information involved in functional balance performance. Still, the approach to combine clinical tests and the use of sensor-tests to address the complex interactions and various dimensions of static and dynamic balance are a potentially valuable prospect for future studies.

## Figures and Tables

**Table 1:** Sensor measurement variables for the Standing balance test (balance) and Three Maximal Chair Stand Test (leg strength).

Test	Measure		Definition	Unit	Algorithm	Reference
Balance (time domain)	NPL	Normalized Path Length	Acceleration ap/ml vector length sum, divided by time.	mg/s	$NPL=\frac{1}{T}\overset{N-1}{\underset{n=1}{\sum }}\left|a_{n+1}-a_{n}\right|$	[25]
	hAREA	Horizontal Sway Area	95% confidence circle area of acceleration	mg²	$hAREA=\pi *\left(hMEAN+1.645*\sqrt{hRMS^{2}-hMEAN^{2}}\right)$	[26,70]
	hRMS	Horizontal Root Mean Square Acceleration	RMS of acceleration in two dimensions (ml and ap)	mg	$hRMS=\sqrt{\frac{1}{N}\overset{N}{\underset{n=1}{\sum }}\left(a_{ml,n}^{2}+a_{ap,n}^{2}\right)}$	[26,70]
	hMEAN	Horizontal Mean Acceleration	Average acceleration in two dimensions (ml and ap)	mg	$hMEAN=\frac{1}{N}\overset{N}{\underset{n=1}{\sum }}\sqrt{a_{ml,n}^{2}+a_{ap,n}^{2}}$	[26,70]
	RMS	Root Mean Square Acceleration	RMS of acceleration, in ml and ap directions separately	mg	$RMS_{d}=\sqrt{\frac{1}{N}\overset{N}{\underset{n=1}{\sum }}\left(a_{d,n}^{2}\right)}$	[26,70]
	P2P	Acceleration Peak to Peak	Acceleration max-min, in ml and ap directions separately	mg	$P2P_{d}=\underset{1\leq n\leq N}{\max}\{a_{d,n}\}-\underset{1\leq n\leq N}{\min}\{a_{d,n}\}$	[25]
Balance (frequency domain)	MDF	Median Frequency	Median of the frequency distribution, in ml and ap directions separately	Hz	$MDF_{d}=\overset{m}{\underset{i=g}{\sum }}SP_{d,i}\geq 0.5\mu_{d,0}$ where m where *𝑚* is the index at which left side becomes bigger than the right side and the spectral moment *μ* is defined by $\mu_{d,k}=\overset{f}{\underset{i=g}{\sum }}{\left(i*\Delta f\right)}^{k}*SP_{d,i}$	[26,70]
	CFREQ	Centroidal Frequency	“Centre” of the frequency distribution, in ml and ap directions separately	Hz	$CFREQ_{d}=\sqrt{\frac{\mu_{d,2}}{\mu_{d,0}}}$	[26,70]
Leg strength	Power Max	Max Vertical Power	Power approximated by mass ∗ vertical acceleration ∗ vertical velocity	W	$PowerMax=\underset{1\leq n\leq N}{\max}\left\{a_{vert,n}*M*v_{vert,n}\right\}$	[34,40]
	VelMax	Max Vertical Velocity	Integral of measured vertical acceleration, compensated for linear drift	m/s	$VelMax=\underset{1\leq n\leq N}{\max}\left\{v_{vert,n}\right\}$	[34,40]
	AccMax	Max Vertical Acceleration	Measured acceleration vertical component	m/s²	$AccMax=\underset{1\leq n\leq N}{\max}\left\{a_{vert,n}\right\}$	[34,40]
	JerkMax	Max Vertical Jerk	Derivative of measured vertical acceleration	m/s³	$JerkMax=\underset{1\leq n\leq N}{\max}\left\{j_{vert,n}\right\}$	[34,40]

ml = medio–lateral direction; ap = anterior–posterior direction; mg = milli gravitational acceleration; N is number of samples in the analysed interval and n the index in interval; T is the total time of the analysed interval; a is the horizontal acceleration vector; d is ml or ap; vert = vertical direction; $a_{vert}\;$ = vertical acceleration; $v_{vert}$ = vertical velocity, defined as the integral of acceleration; $j_{vert}$ = vertical jerk, the symmetric derivative of acceleration; M = body mass; SP = power spectrum; i = the power spectrum index; x = the starting index corresponding to lowest included frequency (0.1Hz); Y = index corresponding to highest included frequency (5Hz); Δf = frequency resolution of the power spectrum.

**Table 2:** Descriptive data and measurements from clinical instruments (n = 31).

Descriptive Data	Value
Age (mean ± sd)	78.7 ± 4.7
Falls last year (n)	10 (32%)
Using a walking aid (n)	9 (29%)
SGPALS active * summer (n)	26 (84%)
SGPALS active * winter (n)	26 (84%)
**Clinical Balance Instruments**, **Median (Q1–3)**	
Mini-BESTest (score)	21 (15–23)
Functional Reach (cm)	22 (18–28)
Modified 4-stage Balance Test (s)	99 (91–120)
Modified Maximal Stepping test (cm) **	67 (58–80) ^†^
**Clinical Leg Strength Instruments**, **Median (Q1–3)**	
Five Times Sit-To-Stand (s)	13.6 (11.6–16.4)
30s Chair Stand Test (n)	11 (9–13)
Sitting leg press 1 RM (1 RM/body weight)	0.98 (0.76–1.32) ^‡^

* SGPALS = Saltin-Grimby Physical Activity Level Scale 1–6, dichotomized into inactive (level 1–2) and active (level 3–6); ** used in correlations for both balance and leg strength instruments; ^†^ 1 missing, ^‡^ n = 21.

**Table 3:** The Spearman’s rho (ϱ) correlation between the clinical instruments.

	MiniB	FR^n^	m4-stageBT	MaxStep^n^	5TSTS	30s CST	1 RM
	Spearman’s Rho (ϱ)						
MiniB	-	0.492 **	0.736 ***	0.769 ***	−0.363 *	0.326	0.190
FR	-	-	0.664 ***	0.609 ***	−0.148	0.145	0.226
m4-stageBT	-	-	-	0.773 ***	−0.447 *	0.390 *	0.422 *
MaxStep	-	-	-	-	−0.547 **	0.574 ***	0.375 *
5TSTS	-	-	-	-	-	−0.777 ***	−0.238
30s CST	-	-	-	-	-	-	0.302
1 RM	-	-	-	-	-	-	-

* significant at 0.05 level (2-tailed); ** significant at 0.01 level (2-tailed); *** significant at <0.01 level (2-tailed); MiniB = Mini-BESTest; FR^n^ = normalized Functional Reach; m4-stageBT = Modified 4-stage Balance Test; MaxStep^n^ = normalized Modified Maximal stepping test; 5TSTS = Five Times Sit-To-Stand; 30 s CST = 30 s Chair Stand Test; 1 RM = One Repetition Maximum.

**Table 4:** Descriptive data for balance sensor tests. Variables are described in terms of the median (Q1–3) n = 31.

Test	Measure	Feet Together	Semi Tandem
Balance (time domain)	NPL (mg/s)	21.1 (17.9–34.4)	36.6 (27.3–60.4)
	hAREA (mg^2^)	8.2 (6.8–14.4)	15.7 (11.4–23.9)
	hRMS (mg)	1.4 (1.2–2.6)	2.8 (2.0–4.2)
	hMEAN (mg)	1.1 (0.9–1.8)	2.3 (1.5–3.4)
	RMS_ap_ (mg)	1.1 (0.7–1.7)	1.8 (1.2–3.3)
	RMS_ml_ (mg)	1.1 (0.8–1.8)	2.0 (1.3–2.9)
	P2P_ap_ (mg)	11.0 (7.7–17.3)	16.9 (12.6–32.0)
	P2P_ml_ (mg)	12.1 (8.5–16.0)	20.1 (11.5–32.4)
Balance (frequency domain)	MDF_ap_ (Hz)	2.3 (2.1–2.6)	2.2 (2.0–2.6)
	MDF_ml_ (Hz)	2.1 (2.0–2.5)	2.2 (1.9–2.4)
	CFREQ_ap_ (Hz)	2.6 (2.5–2.8)	2.6 (2.4–2.9)
	CFREQ_ml_ (Hz)	2.7 (2.5–2.9)	2.7 (2.4–2.7)

For variable abbreviations please see Table 1. ml = medio—lateral movement; ap = anterior—posterior movement.

**Table 5:** Correlation results between clinical balance instruments and sensor-tests for position Feet Together (n = 31), using Spearman’s rho (ϱ).

Test	Measure	MiniB	FR^n^	m4-stageBT	MaxStep^n^
	Spearman’s Rho(ϱ)				
Balance (time domain)	NPL	−0.169	−0.125	−0.125	−0.057
	RMS_ap_	−0.254	−0.221	−0.242	−0.115
	RMS_ml_	−0.264	−0.167	−0.146	−0.109
	P2P_ap_	−0.264	−0.217	−0.258	−0.061
	P2P_ml_	−0.392 *	−0.168	−0.196	−0.119
	hAREA	−0.234	−0.190	−0.173	−0.092
	hRMS	−0.235	−0.182	−0.181	−0.091
	hMEAN	−0.232	−0.208	−0.201	−0.127
Balance (frequency domain)	MDF_ap_	0.156	−0.041	0.177	0.140
	MDF_ml_	0.607 ***	0.351	0.557 **	0.589 ***
	CFREQ_ap_	0.245	0.073	0.247	0.233
	CFREQ_ml_	0.673 ***	0.457 *	0.615 **	0.615 ***

For variable abbreviations, please see Table 1. * correlation is significant at 0.05 level (2-tailed); ** correlation is significant at 0.01 level (2-tailed); *** correlation is significant at <0.01 level (2-tailed); MiniB = Mini-BESTest; FR^n^ = normalized Functional Reach; m4-stageBT = Modified 4-stage Balance Test; MaxStep^n^ = normalized Modified Maximal Stepping test.

**Table 6:** Correlation results between clinical balance instruments and sensor-tests for the Semi Tandem Stance (n = 31), using Spearman’s rho (ϱ).

Test	Measure	MiniB	FR^n^	m4-stageBT	MaxStep^n^
	Spearman’s Rho (ϱ)				
Balance (time domain)	NPL	−0.165	−0.173	−0.190	−0.147
	RMS_ap_	−0.199	−0.267	−0.282	−0.225
	RMS_ml_	−0.167	−0.246	−0.207	−0.132
	P2P_ap_	−0.167	−0.144	−0.235	−0.259
	P2P_ml_	−0.334	−0.304	−0.306	−0.267
	hAREA	−0.186	−0.229	−0.237	−0.195
	hRMS	−0.183	−0.233	−0.238	−0.198
	hMEAN	−0.176	−0.235	−0.239	−0.184
Balance (frequency domain)	MDF_ap_	−0.062	0.208	0.215	0.023
	MDF_ml_	0.032	0.187	0.160	0.070
	CFREQ_ap_	−0.013	0.222	0.221	0.121
	CFREQ_ml_	0.185	0.355	0.284	0.119

For variable abbreviations, please see Table 1. MiniB = Mini-BESTest; FR^n^ = normalized Functional Reach; m4-stageBT = Modified 4-stage Balance Test; MaxStep^n^ = normalized Modified Maximal Stepping test.

**Table 7:** Descriptive data for the leg strength sensor-test (n = 31). Variables are described in terms of the median (Q1–3) values.

Measure	Value
PowerMax (W)	478 (384–531)
VelMax (m/s)	0.6 (0.5–0.7)
AccMax (m/s^2^)	1.8 (1.5–2.2)
JerkMax (m/s^3^)	8.8 (8.1–14.3)

For variable abbreviations, please see Table 1.

**Table 8:** Correlation result between clinical leg strength instruments and the sensor-test for Three Maximal Chair Stand Test (n = 31), using Spearman’s rho (ϱ).

Measure	MaxStep^n^	5TSTS	30sCST	1 RM^n^^†^
	Spearman’s rho (ϱ)			
PowerMax	0.340	−0.414 *	0.561 **	0.523 *
VelMax	0.398 *	−0.366 *	0.639 ***	0.559 **
AccMax	0.529 **	−0.526 **	0.591 ***	0.238
JerkMax	0.526 **	−0.533 **	0.524 **	0.289

For variable abbreviations, please see Table 1. ^†^ n = 21 * correlation is significant at 0.05 level (2-tailed); ** correlation is significant at 0.01 level (2-tailed); *** correlation is significant at <0.01 level (2-tailed).

## Data Availability

The data presented in this study are available on request from the corresponding author. The data are not publicly available due to lack of consent for sharing individual data.

## Supplementary Materials

The following are available online at https://www.mdpi.com/1424-8220/21/5/1765/s1, Figure S1A: Correlations for variables balance sensor-test *Feet Together*, MyBalance prototype concurrent validity testing, and Correlations for variables balance sensor-test *Semi Tandem*, MyBalance prototype concurrent validity testing, Figure S1B: Correlations for variables leg strength sensor-test, MyBalance prototype concurrent validity testing.
