# Supplementary material for: Evaluation of Concurrent Validity between a Smartphone Self-Test Prototype and Clinical Instruments for Balance and Leg Strength
Source: Sensors (Basel). 2021 Mar 4;21(5):1765. doi: 10.3390/s21051765 (PMC7961526; doi:10.3390/s21051765)

Supplementary Figure S1A.

Correlations for variables balance sensor-test *Feet Together*, MyBalance prototype concurrent validity testing.

Correlation Matrix

		NPL_1	apRMS_1	mlRMS_1	apP2P_1	mlP2P_1	hAREA_1	hRMS_1	hMEAN_1	mlTP_1MEAN	apTP_1MEAN	mlMDF_1	apMDF_1	mlCFREQ_1	apCFREQ_1	mlFREQD_1MEAN	apFREQD_1MEAN
NPL_1	Spearman's rho	—															
	p-value	—															
apRMS_1	Spearman's rho	0.921 ***	—														
	p-value	< .001	—														
mlRMS_1	Spearman's rho	0.962 ***	0.866 ***	—													
	p-value	< .001	< .001	—													
apP2P_1	Spearman's rho	0.767 ***	0.915 ***	0.725 ***	—												
	p-value	< .001	< .001	< .001	—												
mlP2P_1	Spearman's rho	0.835 ***	0.806 ***	0.915 ***	0.773 ***	—											
	p-value	< .001	< .001	< .001	< .001	—											
hAREA_1	Spearman's rho	0.970 ***	0.963 ***	0.953 ***	0.864 ***	0.885 ***	—										
	p-value	< .001	< .001	< .001	< .001	< .001	—										
hRMS_1	Spearman's rho	0.977 ***	0.960 ***	0.957 ***	0.844 ***	0.878 ***	0.997 ***	—									
	p-value	< .001	< .001	< .001	< .001	< .001	< .001	—									
hMEAN_1	Spearman's rho	0.987 ***	0.943 ***	0.957 ***	0.781 ***	0.840 ***	0.977 ***	0.984 ***	—								
	p-value	< .001	< .001	< .001	< .001	< .001	< .001	< .001	—								
mlTP_1MEAN	Spearman's rho	0.951 ***	0.867 ***	0.993 ***	0.733 ***	0.918 ***	0.950 ***	0.951 ***	0.947 ***	—							
	p-value	< .001	< .001	< .001	< .001	< .001	< .001	< .001	< .001	—							
apTP_1MEAN	Spearman's rho	0.899 ***	0.990 ***	0.850 ***	0.923 ***	0.815 ***	0.953 ***	0.947 ***	0.927 ***	0.861 ***	—						
	p-value	< .001	< .001	< .001	< .001	< .001	< .001	< .001	< .001	< .001	—						
mlMDF_1	Spearman's rho	-0.096	-0.130	-0.216	-0.175	-0.278	-0.158	-0.152	-0.141	-0.226	-0.152	—					
	p-value	0.608	0.485	0.244	0.347	0.130	0.396	0.414	0.451	0.221	0.414	—					
apMDF_1	Spearman's rho	0.097	0.029	0.038	0.004	-0.080	-0.004	0.003	0.032	0.078	0.026	-0.109	—				
	p-value	0.604	0.878	0.841	0.983	0.669	0.984	0.985	0.862	0.677	0.890	0.559	—				
mlCFREQ_1	Spearman's rho	-0.140	-0.242	-0.215	-0.261	-0.269	-0.218	-0.219	-0.206	-0.231	-0.263	0.844 ***	-0.093	—			
	p-value	0.453	0.188	0.244	0.156	0.143	0.237	0.235	0.264	0.211	0.153	< .001	0.617	—			
apCFREQ_1	Spearman's rho	0.035	-0.050	-0.044	-0.061	-0.187	-0.075	-0.074	-0.038	-0.010	-0.049	-0.004	0.930 ***	0.030	—		
	p-value	0.851	0.789	0.814	0.744	0.313	0.689	0.692	0.839	0.956	0.792	0.984	< .001	0.873	—		
mlFREQD_1MEAN	Spearman's rho	0.094	0.009	0.225	0.015	0.237	0.102	0.107	0.089	0.215	0.004	-0.776 ***	0.007	-0.461 **	-0.053	—	
	p-value	0.614	0.961	0.223	0.937	0.199	0.584	0.564	0.634	0.245	0.985	< .001	0.968	0.010	0.776	—	
apFREQD_1MEAN	Spearman's rho	-0.140	-0.119	-0.116	-0.135	-0.113	-0.103	-0.105	-0.101	-0.140	-0.104	0.169	-0.768 ***	0.176	-0.582 ***	0.015	—
	p-value	0.450	0.521	0.534	0.467	0.544	0.581	0.573	0.588	0.450	0.578	0.362	< .001	0.343	< .001	0.937	—

Note. * p < .05, ** p < .01, *** p < .001

Correlations for variables balance sensor-test *Semi Tandem*, MyBalance prototype concurrent validity testing.

Correlation Matrix

		**NPL_2**	**apRMS_2**	**mlRMS_2**	**apP2P_2**	**mlP2P_2**	**hAREA_2**	**hRMS_2**	**hMEAN_2**	**mlTP_2**	**apTP_2**	**mlMDF_2**	**apMDF_2**	**mlCFREQ_2**	**apCFREQ_2**	**mlFREQD_2**	**apFREQD_2**
NPL_2	Spearman's rho	—															
	p-value	—															
apRMS_2	Spearman's rho	0.966 ***	—														
	p-value	< .001	—														
mlRMS_2	Spearman's rho	0.926 ***	0.875 ***	—													
	p-value	< .001	< .001	—													
apP2P_2	Spearman's rho	0.893 ***	0.941 ***	0.796 ***	—												
	p-value	< .001	< .001	< .001	—												
mlP2P_2	Spearman's rho	0.843 ***	0.804 ***	0.950 ***	0.744 ***	—											
	p-value	< .001	< .001	< .001	< .001	—											
hAREA_2	Spearman's rho	0.965 ***	0.958 ***	0.960 ***	0.912 ***	0.914 ***	—										
	p-value	< .001	< .001	< .001	< .001	< .001	—										
hRMS_2	Spearman's rho	0.969 ***	0.956 ***	0.966 ***	0.902 ***	0.910 ***	0.997 ***	—									
	p-value	< .001	< .001	< .001	< .001	< .001	< .001	—									
hMEAN_2	Spearman's rho	0.987 ***	0.969 ***	0.944 ***	0.901 ***	0.858 ***	0.977 ***	0.983 ***	—								
	p-value	< .001	< .001	< .001	< .001	< .001	< .001	< .001	—								
mlTP_2	Spearman's rho	0.921 ***	0.877 ***	0.996 ***	0.799 ***	0.954 ***	0.963 ***	0.967 ***	0.940 ***	—							
	p-value	< .001	< .001	< .001	< .001	< .001	< .001	< .001	< .001	—							
apTP_2	Spearman's rho	0.966 ***	1.000 ***	0.875 ***	0.941 ***	0.804 ***	0.958 ***	0.956 ***	0.969 ***	0.877 ***	—						
	p-value	< .001	< .001	< .001	< .001	< .001	< .001	< .001	< .001	< .001	—						
mlMDF_2	Spearman's rho	0.110	0.148	-0.043	0.192	-0.069	0.085	0.063	0.091	-0.034	0.148	—					
	p-value	0.556	0.427	0.818	0.300	0.711	0.649	0.738	0.626	0.856	0.427	—					
apMDF_2	Spearman's rho	-0.039	-0.151	-0.105	-0.221	-0.058	-0.138	-0.119	-0.098	-0.120	-0.151	-0.170	—				
	p-value	0.837	0.418	0.574	0.232	0.757	0.458	0.523	0.598	0.519	0.418	0.360	—				
mlCFREQ_2	Spearman's rho	0.162	0.136	-0.025	0.154	-0.073	0.090	0.071	0.096	-0.037	0.136	0.836 ***	-0.040	—			
	p-value	0.383	0.464	0.896	0.406	0.694	0.631	0.702	0.605	0.845	0.464	< .001	0.830	—			
apCFREQ_2	Spearman's rho	-0.174	-0.330	-0.189	-0.402 *	-0.151	-0.289	-0.261	-0.246	-0.200	-0.330	-0.311	0.876 ***	-0.181	—		
	p-value	0.348	0.070	0.308	0.026	0.415	0.115	0.156	0.182	0.278	0.070	0.089	< .001	0.329	—		
mlFREQD_2	Spearman's rho	-0.122	-0.204	-0.052	-0.221	-0.101	-0.138	-0.132	-0.133	-0.095	-0.204	-0.560 **	0.233	-0.319	0.285	—	
	p-value	0.511	0.270	0.781	0.232	0.587	0.458	0.477	0.475	0.609	0.270	0.001	0.206	0.081	0.120	—	
apFREQD_2	Spearman's rho	-0.185	-0.182	-0.068	-0.115	-0.041	-0.137	-0.146	-0.158	-0.075	-0.182	-0.351	-0.543 **	-0.258	-0.337	0.225	—
	p-value	0.319	0.328	0.715	0.540	0.826	0.462	0.433	0.395	0.688	0.328	0.053	0.002	0.160	0.064	0.224	—

Note. * p < .05, ** p < .01, *** p < .001

Supplementary Figure S1B.

Correlations for variables leg strength sensor-test, MyBalance prototype concurrent validity testing.

Correlation Matrix

		PowerMEAN	VelMaxMEAN	AccMaxMEAN	JerkMaxMEAN	STS_DurationMEAN
PowerMEAN	Spearman's rho	—				
	p-value	—				
VelMaxMEAN	Spearman's rho	0.767 ***	—			
	p-value	< .001	—			
AccMaxMEAN	Spearman's rho	0.511 **	0.679 ***	—		
	p-value	0.004	< .001	—		
JerkMaxMEAN	Spearman's rho	0.371 *	0.469 **	0.889 ***	—	
	p-value	0.041	0.008	< .001	—	
STS_DurationMEAN	Spearman's rho	-0.122	-0.211	-0.222	-0.105	—
	p-value	0.512	0.254	0.229	0.573	—

Note. * p < .05, ** p < .01, *** p < .001

Plot

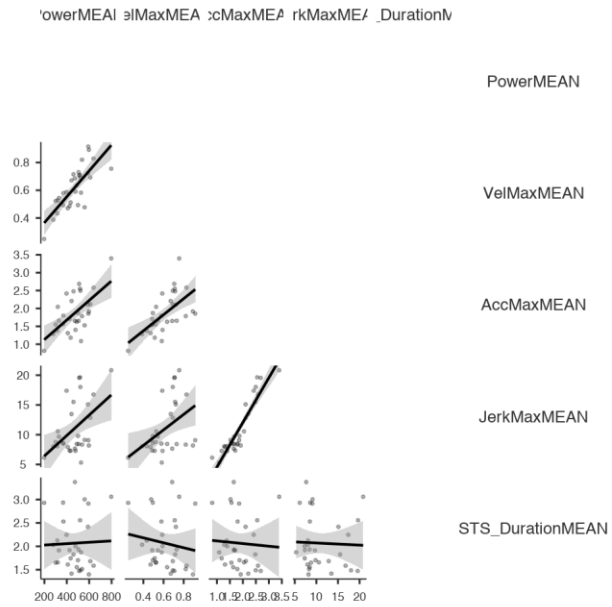

Supplement: Supplementary file 1 [file sensors-21-01765-s001.pdf]
